# 1030. Chasing the Long Tail of Infectious Diseases: Detecting *Capnocytophaga canimorsus* and *Pasteurella multocida* Infections with A Plasma-based Microbial Cell-Free DNA Next Generation Sequencing Test

**DOI:** 10.1093/ofid/ofab466.1224

**Published:** 2021-12-04

**Authors:** Nicholas R Degner, Ricardo Galvan-Castillo, Jose Alexander, Aparna Arun, Christiaan R de Vries, Ann Macintyre, Bradley Perkins, Asim A Ahmed, Matthew Smollin

**Affiliations:** 1 Karius Inc., San Francisco, California; 2 Karius, Inc., Orlando, Florida; 3 Karius, Redwood City, California; 4 Karius, Inc, Redwood City, CA

## Abstract

**Background:**

*Capnocytophaga canimorsus* (*Cc*) and *Pasteurella multocida* (*Pm*) are gram negative bacterial commensal pathogens typically from dogs or cats that can cause severe infection in humans when spread through licks, scratches or bites. The diagnosis of these infections can be limited by: (1) their fastidious nature and difficulty to culture; (2) the nonspecific manifestations of the infections; and (3) the unreliability of dog or cat exposure history. Open-ended microbial cell free DNA (mcfDNA) next-generation sequencing (NGS) offers a potential solution to overcome these limitations.

**Methods:**

The Karius Test^TM^ (KT) developed and validated in Karius’s CLIA certified/CAP accredited lab in Redwood City, CA detects mcfDNA in plasma. After mcfDNA is extracted and NGS performed, human reads are removed, and remaining sequences are aligned to a curated database of > 1500 organisms. McfDNA from organisms present above a statistical threshold are reported and quantified in molecules/µL (MPM). KT detections of *Cc* and *Pm* were reviewed from August 2017 - present; clinical information was obtained with test requisition or consultation upon result reporting.

**Results:**

KT detected 5 cases of *Cc* (25,039 MPM +/- 41,062) and 8 cases of *Pm* (33,264 MPM +/- 69,301) (Table 1). All detections of *Cc* were in adults (60% male) and included 2 cases of culture-negative endocarditis (one with known liver disease) and one case of sepsis with diffuse rash. *Pm* detections occurred in 6 adults and 2 children (75% male) and included 2 cases of culture-negative endocarditis, and single cases each of endovascular graft infection, pneumonia, fever of unknown origin, and a cranial dog bite complicated by an abscess. Two patients had immunocompromising conditions including neuroblastoma and aplastic anemia.

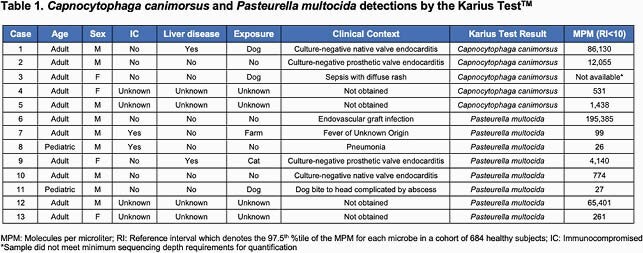

**Conclusion:**

Unbiased, plasma-based mcfDNA NGS provides a rapid, non-invasive test to diagnose diverse clinical infections by *Cc* and *Pm*. These cases highlight the potential of the KT to diagnose infections caused by fastidious/unculturable pathogens with non-specific clinical manifestations and broad differential diagnoses.

**Disclosures:**

**Nicholas R. Degner, MD, MPH, MS**, **Karius Inc.** (Employee, Shareholder) **Ricardo Galvan-Castillo, MD**, **Karius Inc.** (Employee, Shareholder) **Jose Alexander, MD, D(ABMM), FCCM, CIC, SM, MB(ASCP), BCMAS**, **Karius** (Employee) **Aparna Arun, MD**, **Karius** (Employee) **Ann Macintyre, DO**, **Karius, Inc.** (Employee) **Bradley Perkins, MD**, **Karius, Inc.** (Employee) **Asim A. Ahmed, MD**, **Karius, Inc.** (Employee) **Matthew Smollin, PharmD**, **Karius, Inc.** (Employee)

